# Quantitative reactive modeling and verification

**DOI:** 10.1007/s00450-013-0251-7

**Published:** 2013-10-05

**Authors:** Thomas A. Henzinger

**Affiliations:** IST Austria, Klosterneuburg, Austria

**Keywords:** Formal methods, Program verification, Embedded systems, Systems biology

## Abstract

Formal verification aims to improve the quality of software by detecting errors before they do harm. At the basis of formal verification is the logical notion of *correctness*, which purports to capture whether or not a program behaves as desired. We suggest that the boolean partition of software into correct and incorrect programs falls short of the practical need to assess the behavior of software in a more nuanced fashion against multiple criteria. We therefore propose to introduce *quantitative fitness measures* for programs, specifically for measuring the function, performance, and robustness of *reactive* programs such as concurrent processes.

This article describes the goals of the ERC Advanced Investigator Project QUAREM. The project aims to build and evaluate a theory of quantitative fitness measures for reactive models. Such a theory must strive to obtain quantitative generalizations of the paradigms that have been success stories in qualitative reactive modeling, such as compositionality, property-preserving abstraction and abstraction refinement, model checking, and synthesis. The theory will be evaluated not only in the context of software and hardware engineering, but also in the context of systems biology. In particular, we will use the quantitative reactive models and fitness measures developed in this project for testing hypotheses about the mechanisms behind data from biological experiments.

## Introduction

This article describes the goals of the ERC Advanced Investigator Project QUAREM. The project aims at rebuilding a central part of the formal foundation of computing by replacing the classical, boolean notion of *program correctness* with a new, quantitative measure of *program fitness*. In the platonic, boolean world of classical computer science, programs can only be correct or incorrect.^1^ In the real world, one program is often preferred over another, even if both are technically correct (for example, one may be more robust against faulty inputs than the other), or if both are technically incorrect (one may misbehave less often, or less severely, than the other). Such behavioral preferences can be formalized by quantitative measures of fitness between programs and specifications. We believe that by putting the formal modeling of computational processes on a quantitative foundation, we can pave the way for an increased use of such models, not only in software and system development but also in the natural sciences. In particular, in biology the use of computational models for testing mechanistic hypotheses has been hampered by the lack of quantitative measures of fitness between models and experimental data.

### From proving system correctness to measuring system fitness

Discrete thinking has dominated computer science from its very beginnings and is, in fact, what distinguishes computer science from most other engineering disciplines. This is because every digital computational process can be idealized as a discrete system, which transforms inputs to outputs or interacts with other computational processes in a sequence of discrete steps. Moreover, it is tempting to insist that a hardware and/or software system be *correct*, in the sense that it always behaves as desired, for example, by computing the expected results for all permissible inputs. Discreteness implies that even the smallest change in the code can cause a correct program to become incorrect, and vice versa. This crisp view of computational processes as discrete mathematical objects, and correctness as a boolean, two-valued property, has been one of the cornerstones of the field called *formal verification*.

In verification, we are given descriptions of a system *S* and requirement *r*, and a binary correctness relation *S*⊨*r* asserting that the system *S* satisfies the requirement *r*. Already the notation is suggestive of a logical interpretation, namely, that a computational process *S* defines a mathematical structure in which the statement *r* is *true* or *false*. In a less isolated context, we often describe a system at several different levels of detail using, for instance, the code *S* of a program, a UML model *S*′ of the software, a more abstract model *S*″ which specifies the interfaces of various components but not their internals, etc. We are then interested in reasoning chains of the form

$$S \preceq S' \preceq S'' \preceq \cdots \models r$$

where the binary refinement relation ⪯ has the property that if *S*′ satisfies *r*, and *S* refines *S*′, then *S* satisfies *r* as well; in other words, the refinement relation is requirements preserving. In an idealized setting, software development would proceed top-down in a series of refinement steps, from the requirement *r* to the code *S*, and if that is not possible, the code *S* would be verified against the requirement *r*, and the intermediate models can be used to divide the verification problem into subproblems of smaller complexity.

We submit that typical situations that arise in software engineering practice, as well as in the formulation of biological and physical hypotheses using computational models, are fundamentally different from the idealized setting described above. Real-world requirements are rarely complete and real-world programs are rarely correct, not only because of programming errors but also because of unspecified assumptions and the like. Typically, we are given a number of alternative systems, say, *S*_1_,…,*S*_*m*_, and a number of requirements *r*_1_,…,*r*_*n*_ of interest, some of them functional, some of them performance goals and resource constraints, and others about less tangible attributes such as reliability, trustworthiness, and robustness. Then, with respect to each of the requirements, some of the systems may exhibit more desirable behavior than others. Such a “degree of behavioral desirability,” or *fitness*, can be measured by a directed distance function *d* between systems and requirements, and between systems. The classical setting is one of strictly boolean distances, with *d*(*S*,*r*)=0 if *S*⊨*r* else *d*(*S*,*r*)=1, and *d*(*S*,*S*′)=0 if *S*⪯*S*′ else *d*(*S*,*S*′)=1. We suggest that in general distances should be real-valued, as long as they satisfy certain laws such as the triangle inequality

$$d(S,r) \leq d(S,S') + d(S',r)$$

which generalizes the boolean property of requirements preservation by refinement (if *S*′ satisfies *r*, and *S* refines *S*′, then *S* satisfies *r*). Given a computable distance function, an engineer can measure the fitness of alternative system implementations with respect to various requirements, and select an implementation that fits best to her preferences. Similarly, a scientist can measure the fitness of alternative models for a biological or physical phenomenon with respect to various experimental observations, and choose the model that fits best to the data.

### Qualitative reactive modeling and verification

A main motivation behind verification is the double realization that, in theory, it can be very difficult (even technically impossible) to make precise statements about the run-time behavior of programs and, in practice, it is very difficult to write error-free software, even for expert programmers. This motivation has become vastly more urgent with the rise of concurrent computation (first time sharing, then multiprocessors and networks, now multicores and clouds) because concurrency bugs can be extremely subtle and hard to detect by traditional means such as program testing. The behavior of a concurrent process that interacts with other computational processes is captured by a *reactive model*. More generally, reactive modeling is the mathematical formalization of behavior that consists of interdependent discrete events in time; it has a rich theory based on behaviors as infinite computation histories and/or infinite computation trees, with various canonical formalisms for specifying systems (e.g., finite automata, process algebras), requirements (e.g., temporal logics), and refinement (e.g., simulation relations).

Transcending any particular formalism or language, the theory of reactive modeling has introduced several fundamental paradigms whose impact goes far beyond the description of concurrent computational processes, towards offering a universal methodology for complexity management in discrete systems. These paradigms include

*model composition*: which supports the construction of a complex model from standard components that may interact synchronously or asynchronously, while preserving certain conditional properties of the individual components (assume-guarantee compositionality);
*model abstraction*: and its dual, model refinement, which support the description of a complex system at multiple levels of detail while preserving certain properties across different levels;
*model execution*: which allows the simulation of a reactive system by executing a virtual machine whose instructions correspond to atomic steps and may be defined inductively on the model description (structured operational semantics);
*model checking*: which determines the truth values of temporal requirements over reactive models by automatically and systematically exploring the state space of the model;
*model synthesis*: which allows the automatic construction of a finite-state reactive model that satisfies a given requirement or experiment (if such a model exists).

All of these benefits of reactive models have proved useful not only in the design of complex artificial systems, such as hardware and software systems, but also for representing natural systems and testing biological hypotheses [1]. We aim to preserve as many of these benefits as possible when moving towards a quantitative framework for reactive modeling.

Over the past two decades, reactive models have been extended by quantitative aspects, for example, to capture real time and/or probabilistic behavior.^2^ As a result, reactive models have proved useful to analyze not only functional but also timing requirements of computational systems; not only worst-case but also average-case behavior. Yet the resulting theories have usually remained boolean at their core: in real-time verification, it is normally checked whether or not a timed reactive model satisfies a timing requirement; in probabilistic verification, it is normally checked whether or not a stochastic reactive model satisfies a functional (or timing) requirement with a certain probability. Sometimes logical queries are used to obtain quantities (or “parameters”) that identify the boundary between the satisfaction and nonsatisfaction of a requirement. Inherently quantitative interpretations of requirements, while attempted [2, 3], have remained rare and often fall short of important properties such as compositionality. We believe that a radical paradigm shift from boolean to quantitative evaluations of models is needed in order for reactive modeling to reach its full application potential both inside and outside of computer science.

### Quantitative reactive modeling and verification

To systematically rebuild the theory of reactive modeling on a quantitative foundation, we start with the following steps.

*Fitness measures*: We replace boolean-valued correctness relations *S*⊨*r* which state whether a system *S* satisfies a requirement *r*, by real-valued distance functions *d*(*S*,*r*) which measure the fitness of *S* with respect to *r*. Consider, for example, the reactive requirement that every client request is followed by a system response. The system designer may prefer systems that ignore few requests, or issue few unnecessary responses, or issue responses quickly, or any combination thereof, all of which can be measured by appropriate distance functions.
*Approximation measures*: We replace boolean-valued refinement relations *S*⪯*S*′ which state whether a system *S* faithfully implements a more abstract system description *S*′, by real-valued distance functions *d*(*S*,*S*′) which measure differences in the behaviors of *S* and *S*′. For example, distance functions can measure how much the implementation *S* must be changed in order to conform to the specification *S*′, and/or how much *S* can be changed without violating conformance with *S*′; the latter is a measure of *robustness* for the implementation *S*.
*Computing preferences between systems and synthesizing optimal systems*: Given a system *S* and a requirement *r* (or another system description *S*′), qualitative model checking asks if *S*⊨*r* (or *S*⪯*S*′), whereas quantitative model checking requires the (possibly approximate) computation of the distance *d*(*S*,*r*) (respectively, *d*(*S*,*S*′)). Given a requirement *r*, qualitative model synthesis asks for the automatic construction of a system *S* such that *S*⊨*r*, whereas quantitative model synthesis requires the construction of an optimal system *S*, which minimizes the distance *d*(*S*,*r*) among all possible systems *S*. Using quantitative synthesis, we can derive a preferred implementation from all possible implementations of a requirement. In this way, reactive synthesis is lifted from a constraint-solving problem to an optimization problem.

The project is organized in the form of six technical research topics, each of which contributes to the project in three stages: (i) to develop a quantitative theory of reactive modeling and verification; (ii) to develop algorithms and software tools that implement the theory; and (iii) to apply and evaluate the tools on examples from systems engineering and cell biology.

*Topic 1: Quantitative foundations of reactivity*: The theory of the *ω*-regular languages, whose words represent infinite computation histories, provides a semantic foundation for qualitative reactivity. We strive to generalize this theory to several quantitative settings, including *probabilistic languages*, whose words represent probability spaces on computation histories; *discounted languages*, whose words reflect the discounted use of a resource along computation histories, such as time to first failure; and *limit-average languages*, whose words reflect the long-run average use of a resource, such as mean time between failures. Discounted languages, with their emphasis on the finite prefixes of computations, are generalizations of safety properties; limit-average languages, with their emphasis on the infinite tails of computations, are quantitative analogues of liveness properties.
*Topic 2: Defining and computing distance measures*: A canonical structural refinement relation between reactive models is Robin Milner’s simulation relation. We generalize this relation to several quantitative distances, including *simulation failure distance*, which measures the frequency with which a system violates a requirement; *simulation tolerance distance*, which measures the degree to which the system is more constrained than the requirement; and *simulation robustness distance*, which measures the degree to which the system can be relaxed without violating the requirement. Each of these simulation distances define quantitative measures of fitness with respect to both functional and nonfunctional system requirements.
*Topic 3: Composing and refining distance measures*: Assume-guarantee proof decomposition, requirements preserving abstraction and abstraction refinement, as well as symbolic data structures are some of the most important principles for organizing the qualitative analysis of reactive models with large and unbounded state spaces. We generalize these paradigms to the quantitative setting where distances need to be added when composing, and approximated monotonically when abstracting or refining.
*Topic 4: Measuring system robustness and designing robust systems*: The (informal) notion of *robustness* is an important design criterion for artificial systems, as well as a characteristic attribute of many natural systems. One of the main benefits of a quantitative theory of systems is that robustness can be formalized as mathematical continuity, in the sense that a system is robust iff small changes in the input cause only small changes in the output [4]. A variation of this theme is that, in a robust system, few violations of input assumptions should cause only few violations of output requirements. We develop and study such theories of system robustness.
*Topic 5: Quantitative measures in multicore and cloud computing*: We define and evaluate quantitative measures that attempt to capture modern aspects of concurrent computation. For multicore applications, we wish to quantify the performance of various thread synchronization principles, such as the trade-off between fine-grained atomicity, which offers greater flexibility by permitting many interleavings of different threads, and coarse-grained atomicity, which causes lower overheads for context switching. For data center and cloud applications, we want to quantify the resource cost and utilization of various processor assignment and job scheduling policies.
*Topic 6: Quantitative models in systems biology*: While there have been several widely noticed attempts to unleash the enormous potential of reactive modeling in biology, the most commonly used mechanistic models in cell biology are still primitive by the standards of computer science. We believe that a quantitative framework for reactive modeling will not only increase acceptance by biologists, but also provide techniques for measuring the fitness of reactive models with respect to experimental data, and for synthesizing models of maximal fitness. We collaborate with systems and cell biologists to accomplish this task.

## The state of the art

We build on work from many different subdisciplines of computer science. First, there is a large body of mature research in qualitative reactive modeling and verification. Second, the qualitative setting has been extended in several quantitative directions, most notably in the directions of (i) real-time and hybrid systems, (ii) probabilistic systems, and (iii) weighted and resource-constrained systems. Third, the need for quantitative modeling and analysis has always been evident in networking, performance analysis, and reliability analysis. Fourth, also quantitative measures of software quality have long been advocated in software engineering, albeit generally not on a behavioral level. Fifth, formal metrics for measuring distances between process behaviors have been proposed for giving a mathematical semantics to reactive processes and programming languages. Sixth, quantitative objective functions have a strong tradition in game theory in general, and specifically in games that relate to reactive synthesis. Seventh, approaches to quantitative and imprecise reasoning have become central to modern artificial intelligence as alternative to classical logic-based frameworks. Last, we are indebted to the pioneers of applying reactive models to biological systems. We cannot possibly survey the literature and state-of-the-art in all of these areas in detail; the following must therefore be understood as a personal, biased selection of some related work.

*Qualitative reactive modeling, verification, and synthesis*: The main elements of the classical boolean framework that we touch on in this proposal include the theory of *ω*-automata [5], temporal logics [6], simulation relations [7], model checking [8], compositionality [9], abstraction [10], and reactive synthesis [11].
*Modeling time, probability, and cost*: Reactive models and corresponding verification techniques have been extended by quantitative aspects such as (i) transition times [12] and continuous variables [13]; (ii) transition probabilities [14, 15]; and (iii) transition weights [16] that may represent costs, rewards, or the consumption of a resource (e.g., power) [17]. There are theories of timed automata and timed temporal logics; theories of Markov processes and probabilistic temporal logics; theories of weighted automata and corresponding quantitative temporal logics; and many combinations thereof, such as *probabilistic timed automata* [18] and *priced timed automata* [19]. These theories often include composition operations and refinement relations, model checking and synthesis algorithms, and abstraction principles. Yet most remain essentially boolean theories, addressing boolean questions such as the Yes/No question “in a Markov decision process, is there a scheduler which ensures that a certain state is reached with probability 1?,” and parametric questions such as “what is the maximal probability with which a scheduler can ensure that a certain state is reached?” In this project we aim at a theory where properties have quantitative values, rather than boolean values, over systems.
*Performance and reliability analysis*: Quantitative methods lie at the center of performance analysis [20] and reliability analysis [21], especially of computer networks. Most of these methods are based on average-case analysis, e.g., of throughput and quality-of-service. While some reactive models are popular in this context, such as Petri nets, by and large the emphasis is on analytical (usually equational) models rather than operational (i.e., machine-based) models.^3^ We focus instead on executable reactive models of programs and components, and primarily on a quantitative assessment of the worst case, rather than the average case. The reason is that, while probabilistic assumptions about loads are reasonable for networks, probabilistic assumptions about the inputs to individual software artifacts are much harder to justify.
*Metrics in software engineering*: A main motivation for a quantitative theory of systems is to measure alternative implementations against different criteria. This is exactly the raison d’être for *software metrics* [23]. While software metrics measure mostly the software development process and the static complexity of code, our aim is more ambitious: our distances between programs, and between programs and specifications, take into account the dynamic behavior of programs.
*Metrics in process semantics*: While software metrics live at the extreme practical end of computer science, at the extreme theoretical end, there have been attempts to give a mathematical semantics to reactive processes which is based on quantitative metrics rather than boolean preorders [24, 25]. In particular for probabilistic processes, it is natural to generalize bisimulation relations to bisimulation metrics [26, 27], and similar generalizations can be pursued if quantities enter not through probabilities but through discounting [28] or continuous variables [29] (this work uses the Skorohod metric on continuous behaviors to measure the distance between hybrid systems). While all of these theories are close in spirit and inspiring by technique to our objectives, they have had little practical impact. We believe that by *not* starting with inherently quantitative systems such as probabilistic and hybrid systems, which are complex mathematical objects, but by first defining quantitative measures for simpler, qualitative systems and properties such as plain finite automata, we can give new impulses to the quantitative agenda.
*Quantitative objectives in graph games*: Quantitative objective functions, probabilistic strategies, and discounting belong to the standard repertoire of game theory [30]. Reactive synthesis requires the solution of games played on graphs [31], and for such graph games, the quantitative mean-payoff objective has been studied extensively [32]. Our approach builds on quantitative games in two ways. First, we define distances between systems using simulation games with quantitative objectives, such as discounted-sum and mean-payoff objectives. Second, we apply these quantitative measures also to infinite runs of *automata*, which are used to specify requirements and technically represent “single-player” games.
*Formalisms for quantitative and imprecise reasoning*: In artificial intelligence there was a shift from predominantly logical reasoning to predominantly quantitative reasoning, similar to the shift that we now advocate for reactive modeling and verification. In modern AI, probabilistic approaches [33] play a central role; fuzzy logics [34] are used in some engineering applications; and genetic and evolutionary programming rely on quantitative notions such as fitness [35]. We look neither for an “imprecise” nor for a primarily probabilistic theory of reactive modeling, nor do we aim at constructing heuristic or approximate optimization schemes. On the contrary, we try to precisely measure and compute the differences between system behaviors, based on formally stated preferences about quantifiable attributes such as failure rate or response time.
*Reactive modeling in systems biology*: Recently, reactive modeling languages that were originally designed for representing computational and manufacturing/control processes, such as process calculi, Petri nets, statecharts, and hybrid automata, have been used to represent biological networks [36–40]. The benefits of this approach, which was dubbed “Executable Biology,” are summarized in [1]. Encouraged by biologists, computer scientists have also begun to design bio-specific reactive languages [41, 42]. We believe that our quantitative agenda will accelerate progress in this interdisciplinary direction.

## Selected research topics

### Building a quantitative foundation for reactive systems theory

Every behavior of a reactive system is an infinite word whose letters represent observable events. The foundations of reactive models distinguish between the linear-time and the branching-time view [6].

*The linear-time view* In the linear-time view, the set of possible behaviors of a system are collected in a language, which is a set of infinite words. Formally, we consider a *language* over an alphabet *Σ* to be a boolean-valued function *L*: $\varSigma^{\omega}\rightarrow\mathbb{B}$, rather than a set (think of *w*∈*L* iff *L*(*w*)=1), to make the connection to quantitative generalizations self-evident. Such languages, which are infinite objects, can be defined using finite-state machines with infinite runs, so-called *ω-automata*, whose transitions are labeled by letters from *Σ*. For an *ω*-automaton *A*, let *L*(*A*) be the language accepted by *A*. There is a rich and robust theory of finite-state acceptors of languages, namely, the theory of the *ω-regular languages* [5]. In particular, the *ω*-regular languages are closed under boolean operations, and the interesting questions about *ω*-automata—specifically language emptiness, language universality, and language inclusion—can all be decided algorithmically. In the linear-time view, the *language inclusion* question is the basis for checking if a system satisfies a requirement, and for checking if one system description refines another one: given two *ω*-automata *A* and *B*, the system *A* satisfies the requirement *B* (respectively, refines the system *B*) iff *L*(*A*)⊆*L*(*B*).

There are two obvious and popular, but orthogonal, quantitative generalizations of languages. In both cases, the general language inclusion problem is open, i.e., we do not even know under which circumstances it can be decided. As the language inclusion problem lies at the heart of all linear-time verification, this is an obviously unsatisfactory situation. Therefore a natural direction to start building a quantitative theory for reactive modeling is to obtain a better understanding of the quantitative language inclusion problem, in all of its formulations.

*Probabilistic languages*: The first quantitative view is probabilistic. A *probabilistic word*, which represents a behavior of a probabilistic system, is a probability space on the set *Σ* ^*ω*^ of infinite words. We write $\mathcal{D}(\varSigma^{\omega})$ for the set of probabilistic words. A *probabilistic language* is a set of probabilistic words, i.e., a function *L*: $\mathcal{D}(\varSigma^{\omega})\rightarrow\mathbb{B}$. Probabilistic words can be defined by Markov chains, and probabilistic languages by Markov decision processes (MDPs), whose transitions are labeled by letters from *Σ*. MDPs generalize *ω*-automata by distinguishing between nondeterministic states, where an outgoing transition is chosen by a *scheduler*, and probabilistic states, where an outgoing transition is chosen according to a given probability distribution. Unlike in *ω*-automata, the scheduler may in general be probabilistic. Given an MDP *A* and a scheduler, the outcome is a probabilistic word, and by collecting the outcomes of all schedulers in a set, we obtain a probabilistic language *L*(*A*). The language inclusion question for MDPs—given two finite-state MDPs *A* and *B*, is *L*(*A*)⊆*L*(*B*)—is open, even if schedulers are required to be nonprobabilistic and if *B* has no nondeterministic states. A solution is known only for the special case where both *A* and *B* have no nondeterministic states; this special case is the equivalence problem for Markov chains [43].^4^
*Weighted languages*: In the second quantitative view, a language is a function from words to real values. The value $L(w)\in\mathbb{R}$ of a word *w* may measure the cost or resource (e.g., power) consumption of the behavior represented by *w*. Formally, a *weighted language* is a function *L*: $\varSigma^{\omega}\rightarrow\mathbb{R}$. Weighted languages can be defined by *weighted automata* [16], which are finite-state machines whose transitions are labeled by both letters from *Σ* and real-valued weights. When assigning values to words, given a weighted automaton, we must make two decisions: (i) how to aggregate the infinite sequence of weights along a run of the automaton into a single value, and (ii) if the automaton is nondeterministic, how to aggregate the values of all possible runs over the same word. Canonical choices for (i) are *discounted-sum*, *limit-average* (mean payoff), and *energy* (sum) values; a canonical choice for (ii) is to take the supremum of the values of all runs over the same word. We will motivate these choices below. Here it suffices to say that the language inclusion question *L*(*A*)⊆*L*(*B*) for weighted automata *A* and *B* is undecidable in the limit-average and energy cases [45, 46], and open in the discounted-sum case. Solutions are known only for the special case where *B* is deterministic [47].

Consider an infinite sequence of real-valued weights *v*_*i*_, for *i*≥0, along a run of a weighted automaton. To aggregate such an infinite sequence into a single value, one can take the supremum sup_*i*≥0_ *v*_*i*_ (the largest weight that occurs along the run), or limsup_*i*≥0_ *v*_*i*_ (the largest weight that occurs infinitely often), or liminf_*i*≥0_. Note that if all transition weights of an automaton are 0 or 1, then sup corresponds to the finite (reachability) acceptance condition; limsup corresponds to Büchi acceptance, and liminf to coBüchi acceptance. However in a truly quantitative setting, more general, real-valued aggregation functions seem more interesting and useful, and the following two have been studied extensively in game theory.

*Discounted-sum values*: One mechanism for obtaining a finite aggregate value from an infinite sequence of weights is discounting, which gives geometrically less weight to weights that occur later in the sequence. Given a real-valued discount factor *λ*∈(0,1), the discounted-sum value is ∑_*i*≥0_ *λ* ^*i*^⋅*v*_*i*_. Discounted-sum values depend strongly on the initial part of an infinite run, and hardly at all on the infinite tail. In a way, they are quantitative generalizations of *safety properties*. They are useful, for example, to define the time to failure of a system.
*Limit-average values*: Another standard way of obtaining a finite aggregate value from an infinite sequence of weights is averaging, which gives equal weight to all weights that occur infinitely often in the sequence (and no weight to values that occur only finitely often). The limit-average (or *mean-payoff*) value is the limit of the average weights of all prefixes: $\textrm{liminf}_{n\geq 0}\frac{1}{n}\cdot\sum_{0\leq i\leq n}v_{i}$ (under some technical conditions liminf coincides with limsup in this definition). Limit-average values depend only on the infinite tail of a run; they are quantitative analogues of *liveness properties*. They are useful, for example, to define the mean time between failures of a system, or the average power consumption of a system, etc.

There are isolated results [46–48] about the expressiveness, decidability, and closure properties of quantitative languages, in the probabilistic, discounted weight, and average weight cases, but we lack a complete picture and, more importantly, a compelling overall theory, i.e., a quantitative pendant to the theory of *ω*-regular languages. We cannot even be sure that the discounted-sum and limit-average aggregation functions are in any way as canonical as Streett and Rabin acceptance are in the qualitative case. A topological characterization of weighted languages, akin to the topological characterization of safety and liveness as closed and dense sets in the Cantor topology, and to the Borel characterization of the *ω*-regular languages, may be helpful in this regard.^5^

*The branching-time view* Given the wide open situation of the quantitative linear-time view, it is natural to look also at the branching-time view, which is algorithmically simpler in many cases (for example, while language inclusion checking is PSPACE-hard for finite-state machines, the existence of a simulation relation between two finite-state machines can be checked in polynomial time). Topic 2 will therefore explore the pragmatics of a quantitative branching-time approach. However, we also wish to have a compelling quantitative *theory* of branching time. Such a theory is best based on *tree automata* [51]. This is because in the branching-time view, the possible behaviors of a system are collected in an infinite computation tree which, unlike the set (language) of the linear-time view, captures internal decision points of the system. In a tree, the values of different infinite paths can be aggregated in at least two interesting, fundamentally different ways.

*Worst-case analysis*: Similarly to the linear-time case, we can assign to a computation tree the supremum of the values of all infinite paths in the tree.
*Average-case analysis*: We can interpret a computation tree probabilistically, by assigning probabilities to all branching decisions of the system. Since a branching decision often depends deterministically on the (unknown) external input that the system receives at that point, this approach amounts to assuming a probability distribution on input values or, more generally, on environment behavior. Given such a probabilistic environment assumption, we can assign to a computation tree the expected value over all infinite paths in the tree.

There has been little work on probabilistic and weighted tree automata in the context of reactive modeling and verification. For tree automata that accept worst-case and average-case computation trees whose infinite paths have sup, limsup, discounted-sum, and limit-average values, their decidability and closure properties, as well as connections to quantitative temporal logics and model checking, remain to be investigated. The aim is not only to obtain a complete picture but, which is more important from a practical perspective, to find at least one appealing and tractable quantitative setting.

### Defining, computing, and optimizing distance measures between systems

A main practical goal of the project is to augment refinement preorders between reactive processes with directed distances that measure differences in the behavior of the processes. It is tempting to start with processes that contain quantitative information, such as transition probabilities and/or transition weights, which naturally suggest quantitative measurements. Instead, we propose to first define and study several *quantitative* distance measures between purely *qualitative* reactive processes.

The canonical structural refinement relation between reactive models is Robin Milner’s simulation relation [7]. We generalize qualitative simulation between state machines to quantitative distances [52]. Simulation can be viewed as a game between system *A*, the *implementation*, and system *B*, the *specification*, which is played on the product of the two state spaces. In a state pair (*p*,*q*) consisting of implementation state *p* and specification state *q*, first the implementation chooses a successor state *p*′ of *p*, and then the specification must choose a successor *q*′ of *q* such that the transition of the specification *matches* (i.e., carries the same observation/letter as) the transition of the implementation. If the game continues for infinitely many rounds, the specification wins and is said to *simulate* the implementation; in this case, every behavior of *A* is a behavior of *B*, that is, *L*(*A*)⊆*L*(*B*). On the other hand, if the specification cannot match an implementation move, then the implementation wins. (In this case, it may or may not be the case that *L*(*A*)⊈*L*(*B*); in other words, simulation is a sufficient but not necessary condition for language inclusion.) We propose the following original quantitative generalizations of simulation.

*Simulation failure game*: Suppose that at a state pair where the implementation would win the simulation game, i.e., where the specification has no matching move, we allow the specification to “cheat” by choosing a transition that is not permitted by the description of *B*. Moreover, whenever the specification cheats, the implementation receives a payoff of 1 (all other moves carry a payoff of 0). In this new, quantitative game, the implementation tries to maximize the average payoff (i.e., to make the specification cheat as often as possible), and the specification tries to minimize the average payoff (i.e., to cheat as little as possible). The *value* of the game is the maximal average payoff that the implementation can achieve, no matter how the specification plays. If *A* is simulated by *B*, written *A*⪯*B*, then the value of the game is 0, because the specification never needs to cheat. However, if *A*⋠*B*, then the value is a real number that measures a behavioral difference between *A* and *B*. The *simulation failure distance* *d*(*A*,*B*) between two state machines *A* and *B* is the value of the game.
*Simulation tolerance game*: While simulation failure distance measures the distance between an implementation and a specification when there is no simulation relation, simulation tolerance distance measures the distance between *A* and *B* if *A* is simulated by *B*. If *A*⪯*B*, then *A* may have fewer behaviors and be more constrained than *B*; the following quantitative game measures how much more constrained. We invert the simulation game so that in each round, the specification now moves first, and the implementation tries to match the move of the specification. Whenever no match is possible, the specification gets payoff 1, otherwise 0. In this game the specification attempts to maximize the average payoff, i.e., it makes the implementation “cheat” as often as possible. The value of the game, and the resulting *simulation tolerance distance*, measures how much additional behavioral freedom a specification has when compared with an implementation.
*Simulation robustness game*: Another way to define a distance between an implementation *A* and a specification *B* in the case that *A* is simulated by *B*, is to measure how much the implementation may deviate from its description *A* without violating the property that *A* is simulated by *B*. This is a measure of robustness of the implementation *A* with respect to the specification *B*. Roughly speaking, in the simulation robustness game, the implementation is permitted to “cheat” and receives payoff 1 each time it cheats and the specification can match the move, payoff −∞ if the specification cannot match the move of the implementation (whether or not it is a cheating move), and payoff 0 otherwise. The implementation tries to maximize the average payoff. The value of the resulting game is called *simulation robustness distance* between *A* and *B*.

We defined all three quantitative versions of the simulation game as games with *mean-payoff* objectives [32]. Other variations are possible, for example, discounted versions where cheating is worse the earlier it comes in the game, or lexicographic combinations of qualitative and quantitative objectives [53]. Furthermore, the precise definitions of these games depend on which kind of “cheating” is permitted for the players, e.g., may they consume an arbitrary letter and jump to an arbitrary state, or only change the letter on an existing transition, or consume any letter but not change state, etc. Finally, if the implementation and/or specification contain fairness assumptions, then corresponding variants of the *fair simulation game* [54] need to be considered.

There are even more possibilities for defining distances when the system descriptions contain time stamps (or clock constraints) [55], probabilities [26], and/or costs. In contrast to most semantic work on process metrics that can be found in the literature, our primary concerns are *computational* (i.e., algorithmic): we wish to find, within the large space of possibilities, meaningful distances that (i) can be computed, or at least effectively approximated and/or compared, and (ii) can be used to synthesize optimal systems from requirements. In other words, we want to solve the following two basic problems:

*Quantitative verification*: Given two systems *A* and *A*′, and a requirement *B*, is *d*(*A*,*B*)≤*d*(*A*′,*B*)?
*Quantitative synthesis*: Given a requirement *B*, construct a system *A* such that *d*(*A*,*B*)≤*d*(*A*′,*B*) for all systems *A*′.

Consider, for example, the temporal requirement that every request *a* must be followed by a response *b*, written in temporal logic as □(*a*⇒◊*b*). Classical reactive synthesis uses graph games with qualitative objectives to automatically construct a finite-state machine that satisfies such a requirement [11]. But there are many correct implementations, and we have no control over which implementation is produced by the synthesis algorithm. In quantitative synthesis, by contrast, we specify a distance between any possible implementation and the requirement, and the algorithm is obliged to produce an implementation with minimal distance from the requirement. For the request-response example, we may be interested in the following quantitative criteria for synthesis, among many others:

- Minimize the *maximal* time between requests and responses, or minimize the maximal number of responses between requests. (Note that it is not required that there is any response between two consecutive requests, but on the other hand, an implementation may have many “unnecessary” responses between two consecutive requests.) These objective functions can be expressed using weighted *sup* automata (cf. Topic 1).
- Minimize the *average* time between requests and responses, or minimize the average number of responses between requests. (Note that “average” does not refer to any probabilities, but to the mean of the possibly infinite number of response times along a run.) These objective functions can be formalized using weighted *limit-average* automata (cf. Topic 1).
- Minimize the *expected* maximal (or average) time between requests and responses, or the expected maximal (or average) number of responses between requests. Such objective functions can be stated only in a branching-time framework, relative to a probabilistic assumption about how the environment produces requests (again, cf. Topic 1).

Each criterion leads to a different preference order between systems, and to a different optimal implementation. Such optimal implementations can be synthesized automatically by solving graph games with suitable quantitative objectives. We have already solved specific instances of the quantitative synthesis problem [56, 57], but many settings remain to be investigated.

A particularly intriguing aspect of a quantitative framework is that it permits the synthesis of implementations from inconsistent or incompatible requirements [58]. Even if there is no solution to the synthesis problem which satisfies all requirements in the boolean sense, we may still be interested in a solution that comes as close as possible to satisfying the requirements.

### Composing and refining distance measures between systems

It has been a long, and still incomplete, road from the principle of (qualitative) reactive modeling to the practice of reactive verification. Some critical milestones along this way include symbolic state-space exploration [8], assume-guarantee decomposition of model-checking tasks [9], and counter-example guided abstraction refinement [59]. There is every reason to expect that in order for a quantitative framework to be of practical use, it must admit the *symbolic computation* of distances, *compositional reasoning* about distances, and the *abstraction/approximation* of distances. In addition, it is of course desirable to build a quantitative framework in such a way that the boolean case becomes a special case of the quantitative case. In the past, data structures for symbolic reasoning about times and probabilities have received much attention [18, 60], so we concentrate here on compositional and abstract reasoning with quantities.

*Quantitative compositional reasoning*: In the boolean setting, the basic compositional inference rule states that *A*_1_⪯*B*_1_ and *A*_2_⪯*B*_2_ together imply *A*_1_∥*A*_2_⪯*B*_1_∥*B*_2_. This rule is of practical importance because it ensures that the refinement between two complex systems *A*_1_∥*A*_2_ and *B*_1_∥*B*_2_ can be proved component by component. A quantitative analogue of the rule is
  $$d(A_1\parallel A_2,B_1\parallel B_2) \leq f(d(A_1,B_1),d(A_2,B_2))$$
  for a suitable function *f*, such as addition [61]. More interesting is assume-guarantee compositionality, which in the boolean setting states that *A*_1_∥*B*_2_⪯*B*_1_ and *B*_1_∥*A*_2_⪯*B*_2_ together imply *A*_1_∥*A*_2_⪯*B*_1_∥*B*_2_. This inference rule is stronger than the basic compositional rule because to establish the premises, it suffices to prove that *A*_1_ refines *B*_1_ *under the assumption* that the inputs of *A*_1_ are constrained by the environment *B*_2_, and that *A*_2_ refines *B*_2_ under the environment assumption *B*_1_. The assume-guarantee rule holds for certain probabilistic systems [62], but it is open how quantitative assume-guarantee reasoning can look like in general.
*Quantitative abstract and approximate reasoning*: Abstraction is perhaps the most vexing problem and greatest challenge in quantitative settings. Abstraction is a powerful mechanism for relating models of different precision [10]. However, in the boolean setting, the theory of abstraction is different from theories of approximation. A theory of approximation measures the precision of a model in terms of an error bound on how much a model may deviate from the system, and such error bounds are usually quantitative. For example, while discounted-sum automata cannot be determinized precisely, they can be determinized approximately [63]. A theory of abstraction, by contrast, measures the precision of a model in terms of which properties of the system are preserved in the model. Consider, for example, the property that event *a* is never followed by event *b*. A model may preserve such a property despite possibly introducing a large error. On the other hand, even a model that introduces only a small error may violate the property when the system does not. The preservation of properties ensures that it suffices to check a property on a simple, abstract model in order to prove the property for a complex system. In the quantitative setting, we have no satisfactory, general theory of abstraction, and it is likely that in order to obtain such a theory, it needs to be combined with a theory of approximation. Also, one might envision that a generalized triangle inequality
  $$d(A,B) \leq g(d(A,A'),d(A',B))$$
  (for some function *g*) can form the basis for quantitative reasoning with models of different precision: in order to bound the distance between a model *A* and a requirement *B*, it should suffice to compute the distance between *A* and a more abstract model *A*′, and the distance between *A*′ and *B*.

A significant benefit of abstract models is that they can often be refined automatically exactly to the precision that is needed to prove a desired property [59]. Such automatic abstraction refinement lies at the heart of modern software model checking [64] and has been pursued also for probabilistic software [65]. In a quantitative setting, abstraction refinement can be used to over- or underapproximate the underlying, concrete values of individual runs, with monotonically increasing precision [66]. This supports *anytime verification*, where additional refinement steps give better and better estimates of, say, the resource consumption along a run. The ultimate goal is to develop a comprehensive system design methodology based on quantitative reactive models which makes use of composition, approximation, abstraction refinement, and the interplay of these operations.

### Measuring system robustness and synthesizing robust systems

A universal challenge in systems design is the construction of systems whose behavior is robust in the presence of perturbations. While robustness is understood for most physical engineering artifacts, and increasingly also for biological systems [67], it is rarely heard of in connection with software. This is because computer programs have traditionally been idealized as discrete mathematical objects. We often lose sight of the fact that the mathematical representation of a software system is just a model, and that the actual system executes on a physical, imperfect platform and interacts with a physical, unpredictable environment. This physicality has its extreme manifestation in embedded control systems (say, of aircraft), yet many traditional models for timed, hybrid, and probabilistic systems do not even take into account that in reality, event times and sensor input values can be measured only with finite precision, and most probabilities can only be estimated with large tolerances (if at all). This is why the *robustness* of reactive systems has been recognized as an important concern for some time now [68]. However, the few, mostly theoretical approaches towards designing robust software systems [69] remain unconvincing and have had little practical impact.

One of the advantages of a quantitative setting is that we can formalize robustness as mathematical continuity [4]: a system is *continuous* if continuous changes in the environment (or execution platform) cannot cause discontinuous changes in the system behavior. For a continuous reactive system, for every positive real epsilon there must be a positive real delta such that delta changes in the input values and input times cause at most epsilon changes in the output values and output times. For example, a system that reads a sensor value, adds a constant, and outputs the result after some constant time delay, behaves continuously. On the other hand, a system that checks if the sensor value is greater than a threshold, and depending on the result of the check, invokes two different control algorithms with two different execution times to compute the output, does not behave continuously. A preferred, continuous way to combine the two control algorithms would be to execute both procedures if the input value is near the threshold, interpolate the result, and release the output after a predetermined delay. There has been recent work on checking the continuity of systems [70, 71], but we know of no systematic design or synthesis methodology for continuous systems.

Another kind of robustness is resilience against faulty environment assumptions. Assumptions about the environment can be wrong because the environment may change, there may be malicious intent, or the specifier may simply have incomplete or erroneous information about the environment of a system. Traditionally, if the environment violates an assumption, then by definition, a system satisfies any requirement, no matter what it does, because falsehood on the left-hand side of an implication makes the implication true. Yet we wish robust systems to behave “reasonably,” and degrade gracefully, even if the environment misbehaves. For safety properties, we can quantitatively measure this kind of robustness as follows. Whenever the environment violates its safety assumption, by extending a safe finite behavior with an unsafe event/letter, we count an environment error. Similarly, whenever the system violates its safety requirement, we count a system error. The smaller the limit of the ratio of environment to system errors, the more robust is the system. In such a quantitative framework, we can synthesize robust systems by solving so-called *ratio games* [72], an approach that remains to be extended to general liveness properties.

### Quantitative measures in multicore and cloud computing

Concurrent programming is the area of software development that is most prone to programming errors, yet least amenable to testing, because the behavior of concurrent programs is nondeterministic and therefore irreproducible. Concurrent software is thus a prime candidate for formal verification; it is also a prime application area for reactive modeling because the components of a concurrent program (threads, actors, tasks) interact with each other. In addition, there is tremendous urgency because concurrent software is becoming ubiquitous both in the small, on multicore processors, and in the large, in data centers. New programming paradigms, such as software transactions [73] for small-scale concurrency and map-reduce [74] for large-scale concurrency, are among the most discussed topics in computing today. It is therefore only natural that we target concurrent software, both in the small and in the large, as a testing ground for our quantitative agenda. We will do so by developing and evaluating quantitative performance and cost measures for thread concurrency on multiprocessors, and for job and task concurrency in cloud computing.

*Performance metrics for shared-memory concurrency*: There are at least two compelling needs for such metrics. First, a program, such as a new implementation of a concurrent data structure or transaction manager, is developed on, say, an 8-core machine but will run, in the future, on 16, 32, and more cores. We need performance metrics that can predict how concurrent programs scale to an ever increasing number of cores. Second, synthesis methods are used to add synchronization constructs, such as locks and/or fences, to buggy concurrent programs in order to prevent incorrect interleavings of threads [75, 76] and to structure legacy software into atomic transactions [77]. We need performance metrics that can predict how these synchronizations should be placed in order to minimize damage to performance. The measures that are currently used are simple—such as the number and size of atomic sections, the number of fences, the number of variables or objects that are locked at any given time—but it is unclear how predictive these measures are. It is also unclear to which degree architectural aspects, such as caching, need to be taken into account; they play an important role, for example, in execution-time analysis [78].
*Cost metrics for data center and cloud computing*: Cloud computing aims to give users virtually unlimited pay-per-use computing resources without the burden of managing the underlying infrastructure [79]. We believe that, in order to realize the full potential of cloud computing, the user must be presented with a pricing model that offers flexibility at the requirements level, such as a choice between different completion times and different levels of fault tolerance, and the cloud provider must be presented with a programming model that offers flexibility at the execution level, such as a choice between different processor assignment and scheduling policies. In such a flexible framework, with each job, the user purchases a virtual computer with the desired speed, reliability, and cost characteristics, and the cloud provider can optimize the utilization of resources across a stream of jobs from different users. To implement such a framework, we need quantitative measures that quickly and reliably estimate trade-offs between deadlines, fault protection, and resource consumption for networks of interacting tasks.

In both the multicore and cloud scenarios, theoretical performance and cost measures need to be evaluated experimentally, by building both simulators and prototype systems. For the measures that, according to the evaluation, are most accurate in predicting performance and/or resource needs, one may develop game-based algorithms for synthesizing systems that are optimal with respect to the considered measures [80]. Such algorithms can be then used for *optimal lock synthesis*, to add locks and other synchronization constructs to thread concurrent code, and for *optimal schedule synthesis*, to assign the tasks of a job to the processors of a data center.

### Quantitative models in systems biology

An ultimate measure of success for a scientific paradigm is whether it proves useful outside of the field in which it originated. Reactive modeling has the potential for such a success: as the theory behind describing interacting discrete processes using syntax that can be executed, composed, and refined, reactive modeling is useful wherever a dynamical system is, at some level of granularity, best viewed as a system of discrete, causally related events. This, for example, is the case with metabolic pathways in the cell, and with other biological networks [1].

In this project we will not primarily focus on designing new reactive modeling languages or modeling styles for describing certain biological and/or biochemical phenomena (although this would be a welcome side-effect), but we plan to explore and increase the verification and analysis benefits that become available when reactive models are used in biology. In particular, we will pursue the following two directions in close collaboration with experimental systems biology groups, which will supply the biological data.

*Quantitative measures of models and data*: Experiments generate large amounts of data, and in cell biology, a reactive model represents a hypothesis about the mechanism behind the collected data. The hypothesis can be validated by measuring the fit between the model and the data. In other words, for a biological model, the data that results from experiments plays the same role that, for a software model, is played by the requirements. We plan to use the quantitative techniques developed in this project to measure the fit between reactive models and biological data, and to automatically synthesize best-fitting reactive models from data.
*Quantitative techniques for state-space exploration*: A second way for validating a reactive model of a biological network is to use the model for predicting the outcome of new experiments, and then compare the predicted outcomes against the observed outcomes. For such predictions, one may execute, or *simulate*, the model [81]; one simulation run corresponds roughly to one “in-silico” experiment. This, however, is a very inefficient way for analyzing a model [82], akin to obtaining information about a software system solely by testing the program. A main benefit of reactive models in software is that they can be analyzed by state-space exploration techniques (so-called *model checking*), and the same benefit is offered by reactive models in biology [83]. However, the state spaces of biological systems are usually unbounded, and their transitions probabilistic. This calls for the adaptation of proven state-space exploration techniques, and the development of new techniques and heuristics that are specifically targeted towards biological and biochemical systems. We have started to design such techniques—including on-the-fly state-space generation, abstraction, and abstraction refinement—for continuous-time Markov models of (bio)chemical reactions [84, 85]. Also techniques from hybrid systems show great promise, such as switching between discrete and continuous variable representations depending on the population counts for different molecules/species.

Our aim is to demonstrate that the benefits of using quantitative reactive models in biology and other sciences are not limited to the observation that these models can naturally and unambiguously express mechanistic hypotheses, but that they also can come with a set of computational analysis techniques and tools that are far more powerful than simulation.

## Summary

The *high-level objective* of this project is to provide, as more nuanced alternative to the classical *boolean framework* of reactive modeling and verification, a *quantitative framework*. The boolean framework is based on binary satisfaction relations between reactive systems and behavioral requirements, and on binary refinement relations between reactive systems. A fully quantitative framework ought to be based on directed distances between systems which measure differences in their behavior, and directed distances between systems and requirements which measure the fitness of a system with respect to a requirement.

The *practical objective* is to increase the appeal and scope of reactive modeling and verification techniques. Reactive models have already proved their usefulness in many fields of engineering, and recently also in the natural sciences, specifically in cell biology [1]. Yet reactive modeling and verification techniques have also encountered limits and revealed practical limitations of the boolean framework. A quantitative framework will give a new impetus to reactive modeling and verification, both inside and outside of computer science, and open new perspectives and applications for the reactive approach.

The *theoretical objective*, and main challenge, of the project is to provide within a quantitative framework many of the paradigms that have made the boolean framework appealing. These include modeling paradigms such as compositionality and abstraction refinement, and verification paradigms such as model checking and reactive synthesis. A quantitative framework offers special promise for synthesis, where one naturally desires to synthesize, from behavioral specifications, implementations that are optimal according to a chosen metric.

We have outlined several concrete challenges that need to be overcome on the way towards a comprehensive quantitative theory for reactive modeling and verification. While we do not expect that all of the problems we discussed can be solved, we are confident that some of them will be solved, will lead to new questions, and ultimately to quantitative methods of practical usefulness. We have also presented two application areas that we plan to pursue in order to evaluate the quantitative techniques and tools that will result from this project, one in concurrent computing and the other in systems biology. We believe that quantitative thinking about the reactive modeling and analysis of systems will cause a paradigm shift regarding how these techniques are viewed and when they are used, especially if they are supported by a powerful theory and easy-to-use software tools.

If successful, the project has the potential to significantly increase the use of reactive modeling and verification techniques in hardware and software engineering, and also in the computational exploration of mechanistic models for biological systems. While current verification technology is limited to checking reactive models of moderate size against perfectly specified design requirements, a successful project will allow us to compute, for a given model, quantitative measures of fitness with respect to a wide variety of design criteria such as functionality, reliability, performance, robustness, and cost. The computed measures can provide quantitative information to the system designer when choosing a system architecture and/or implementation. Second, a successful project will allow us to guide the automatic synthesis of a hardware or software component from a reactive specification towards an implementation that is optimal with respect to the specified criteria. Third, for scientists that use reactive models to explore mechanisms behind biological phenomena, a successful project will allow them to identify the modeling hypotheses that best fit the experimental data.

## Footnotes

1 The platonic view of programs as (purely) mathematical objects has a long and storied tradition in computer science, going back to John McCarthy, Tony Hoare, Edsger Dijkstra, and many others.

2 Sometimes the very term “quantitative modeling” is used synonymously with probabilistic modeling, and within probabilistic analysis, the term “quantitative” distinguishes reasoning about general probabilities from reasoning about 0 and 1 probabilities. We view probabilistic models as one (important) kind of quantitative models, but other, nonprobabilistic quantitative measures can be useful for modeling real-valued system attributes such as resource consumption and reliability.

3 Recent efforts have built bridges between verification and performance analysis [22].

4 Even in the absence of nondeterminism, some questions about finite generators of probabilistic words (Rabin’s “probabilistic automata”) are undecidable [44].

5 While probabilistic, discounted-sum, and limit-average values are real-valued, there have also been integer-valued attempts at classifying weighted languages. They often focus on the summation of the weights along a run, by considering either finite runs [16] or upper and lower bounds on sums of both positive and negative weights (so-called *energy* values) [17]. The theory of *regular cost functions* abstracts quantitative values, such as infinite sums, to the two boolean values *bounded* and *unbounded* [49]. Another approach uses write-only registers to compute values [50].
